# THE LONGITUDINAL EFFECTS OF PERSISTENT PAIN AND SLEEP HEALTH ON FRAILTY AMONG COMMUNITY-DWELLING OLDER ADULTS

**DOI:** 10.1093/geroni/igae098.3528

**Published:** 2024-12-31

**Authors:** Tuo Yu Chen, Philile Mgabhi, Feng-Jen Tsai, Chyi-Huey Bai, Hui-chuan Hsu, Yi-Hua Chen, Yi-Chun Kuan

**Affiliations:** Taipei Medical University, Taipei, Taipei, Taiwan (Republic of China); Taipei Medical University, Taipei, Taipei, Taiwan (Republic of China); Taipei Medical University, Taipei, Taipei, Taiwan (Republic of China); Taipei Medical University, Taipei, Taipei, Taiwan (Republic of China); Taipei Medical University, Taipei, Taipei, Taiwan (Republic of China); Taipei Medical University, Taipei, Taipei, Taiwan (Republic of China); Shuang Ho Hospital, New Taipei, New Taipei, Taiwan (Republic of China)

## Abstract

Research has shown that persistent pain can affect frailty of community-dwelling older adults. Additionally, evidence supports the significant association of sleep health with persistent pain and frailty. However, the relationship between persistent pain, sleep health, and frailty is not yet fully understood, and it remains unclear whether better sleep health could alleviate the impact of persistent pain on frailty. We investigated whether baseline sleep health and persistent pain predicted frailty at 4-year follow-up among community-dwelling older adults. Besides, we examined whether the relationship between persistent pain and frailty differed by sleep health. Data were from the Taiwan Longitudinal Study on Aging (N=2,562;2011-2015). A frailty index was built using 37 items on functioning and physical and mental health. Persistent pain was measured with a 5-level categorical variable (no pain [reference], mild pain, mild persistent pain, moderate persistent pain, severe persistent pain). Sleep health was assessed using the SATED model (satisfaction-alertness-timing-efficiency-duration). Covariates included baseline sociodemographic status (age,sex,education), errors in cognitive tests, frailty, and poor health behaviors (drinking,lack of exercise). Results showed that baseline moderate persistent pain (OR=1.44,95%CI=1.03-2.01), severe persistent pain (OR=1.65, 95%CI=1.06-2.57), and poor sleep health (OR=1.14,95%CI=1.04-1.26) increased the risks of frailty at 4-year follow-up. A significant synergistic effect between persistent pain and sleep health was observed, such that the risks of frailty were reduced among individuals with better sleep health, even with more severe persistent pain. Overall, persistent pain and sleep health predicted frailty. Improving sleep health among older adults with persistent pain may help mitigate the risks of frailty.

